# Supplementary material for: Splice-Junction-Based Mapping of Alternative Isoforms in the Human Proteome
Source: Cell Rep. Author manuscript; Available in PMC 2020 Jan 15. (PMC6961840; doi:10.1016/j.celrep.2019.11.026)
Supplement: 3 [file NIHMS1546469-supplement-3.zip › DF2/PXD0010154/Testis-90-Q9NQC7-ACVESTILLHINDIIPESVTQER.pdf]

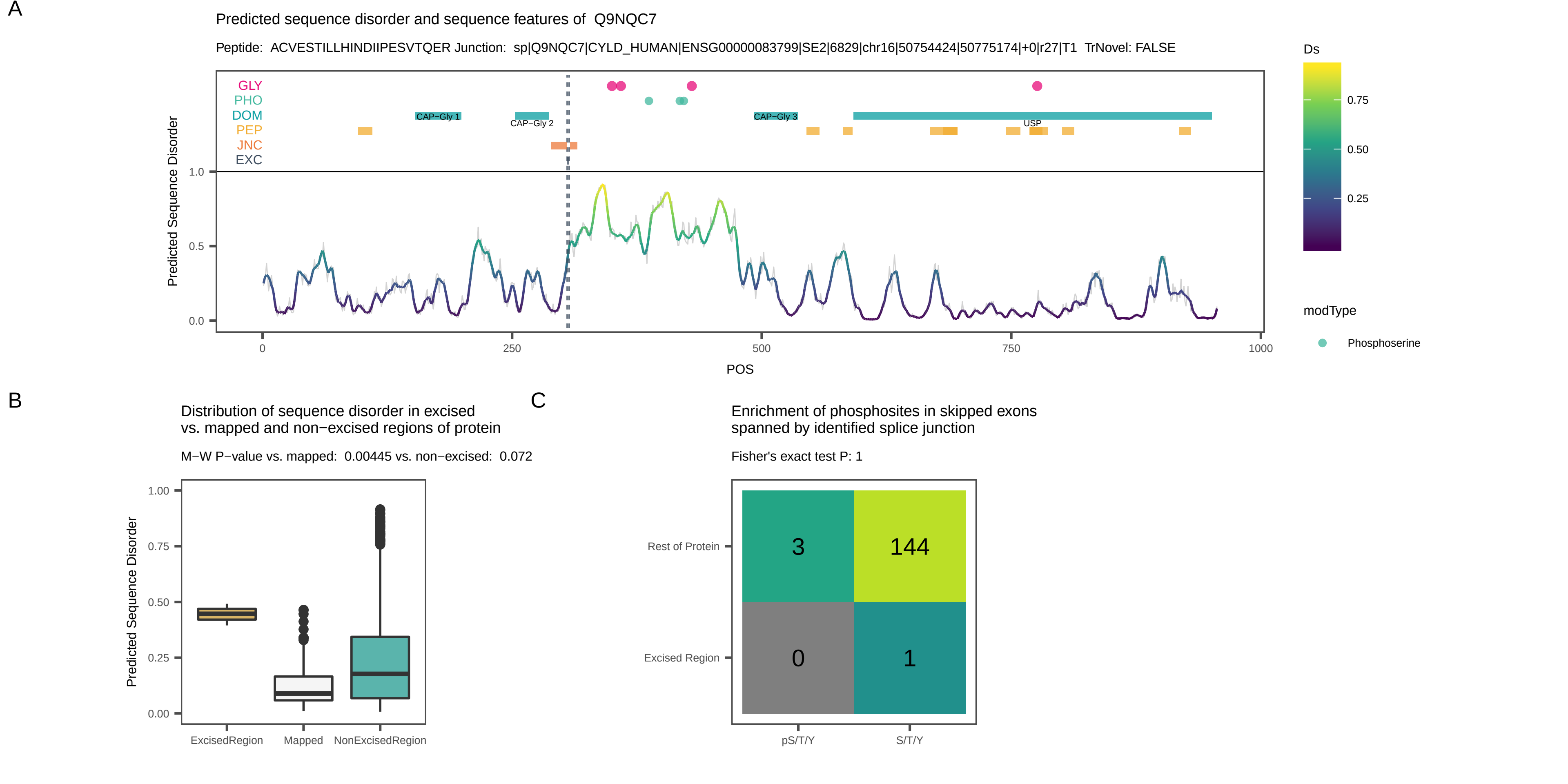
A
Predicted sequence disorder and sequence features of Q9NQC7
Peptide: ACVESTILLHINDIIPESVTQER Junction: sp|Q9NQC7|CYLD_HUMAN|ENSG00000083799|SE2|6829|chr16|50754424|50775174|+0|r27|T1 TrNovel: FALSE
GLY
PHO
DOM
PEP
JNC
EXC
CAP−Gly 1
CAP−Gly 2
CAP−Gly 3
USP
Predicted Sequence Disorder
1.0
0.5
0.0
0
250
500
750
1000
POS
Ds
0.75
0.50
0.25
modType
Phosphoserine
B
Distribution of sequence disorder in excised vs. mapped and non−excised regions of protein
M−W P−value vs. mapped: 0.00445 vs. non−excised: 0.072
Predicted Sequence Disorder
1.00
0.75
0.50
0.25
0.00
ExcisedRegion
Mapped
NonExcisedRegion
C
Enrichment of phosphosites in skipped exons spanned by identified splice junction
Fisher's exact test P: 1
Rest of Protein
Excised Region
3
144
0
1
pS/T/Y
S/T/Y
